# Serum Amyloid A Level in Egyptian Children with Familial Mediterranean Fever

**DOI:** 10.1155/2016/7354018

**Published:** 2016-12-13

**Authors:** Hala M. Lofty, Huda Marzouk, Yomna Farag, Mohammad Nabih, Iman A. S. Khalifa, Noha Mostafa, Ahmed Salah, Laila Rashed, Kamal El Garf

**Affiliations:** ^1^Department of Pediatrics, Faculty of Medicine, Cairo University, Cairo, Egypt; ^2^Department of Medical Biochemistry, Faculty of Medicine, Cairo University, Cairo, Egypt; ^3^Department of Rheumatology, Faculty of Medicine, Cairo University, Cairo, Egypt

## Abstract

*Background and Objectives*. SAA is an acute-phase reactant detected during an FMF attack or other inflammatory conditions. High SAA levels may increase the risk of amyloidosis. The aim of the study is to measure the serum amyloid A (SAA) level in a group of Egyptian children with familial Mediterranean fever (FMF) and study its various correlates, if any.* Methods*. The study enrolled seventy-one children with FMF.* Results*. SAA level was high in 78.9% of the studied patients with a mean of 81.62 ± 31.6 mg/L, and CRP was positive in 31% of patients. There was no significant releation between SAA level and any demographic or clinical manifestation. High SAA was more frequent in V726A allele (16.9%) followed by M694V allele (12.3%). Elevated SAA levels were more frequent in patients on low colchicine doses. Forty-five percent (45%) of patients have low adherence to colchicine therapy.* Interpretation and Conclusion*. High SAA levels were detected two weeks after last FMF attack in a large percentage of Egyptian FMF children. This indicates that subclinical inflammation continues during attack-free periods, and SAA could be used as a marker of it.

## 1. Introduction

Familial Mediterranean fever (FMF) is the most common inherited autoinflammatory syndrome, with an autosomal-recessive pattern of inheritance [1]. The highest prevalence is among Turks, Arabs, Jews, and Armenian populations [2]. It is caused by Mediterranean fever (MEFV) gene mutation mapped onto the short arm of chromosome 16 [3, 4].

FMF is characterized by recurrent self-limited episodes of fever, peritonitis, arthritis, pleuritis, and erysipelas-like erythema [5, 6]. The clinical attacks are accompanied by elevation of erythrocyte sedimentation rate (ESR) and acute-phase reactants as C-reactive protein (CRP), serum amyloid A (SAA), and fibrinogen. These entire laboratory parameters usually return to normal in the attack-free periods [7]. However, it has been reported that subclinical inflammation may continue during the attack-free periods in some patients and may lead to the development of amyloidosis [8, 9] that maybe complicated by end-stage renal disease [10].

Amyloidosis is not necessarily related to the severity of the disease, as it may occur in patients with mild and infrequent attacks, as well as in asymptomatic patients who do not have any clinical manifestation of FMF (phenotype II). However, high levels of SAA, which is the precursor of the main protein related to the amyloid deposit in FMF, for prolonged duration are mandatory for the development of amyloidosis [11, 12].

Colchicine, the main line of treatment in FMF, is effective in preventing attacks in 60–70% of patients. However, 20–30% of patients respond only partially to maximal tolerated doses of colchicine and 5% of patients are nonresponders. The dose of colchicine in children ranges between 1 and 2 mg/day, guided by response and tolerability [13, 14]. However, SAA may be elevated in some patients in attack-free periods. In such patients, an increase in colchicine dose was reported to result in further decrease in SAA level [12, 15], thereby limiting the development of amyloidosis. Some studies reported poor compliance to colchicine in 40% of patients, which may be a contributing factor to colchicine nonresponse, requiring more efforts to educate patients and their caregivers and to solve any problems leading to this incompliance [16].

It was, therefore, intriguing to measure the SAA levels in Egyptian FMF children two weeks following the last attack and study their various correlates and value in detecting subclinical inflammation and rates of incompliance and plan for management.

## 2. Material and Methods

The study included 71 children with FMF, 36 males and 35 females. Patients were diagnosed according to the new pediatric FMF criteria [6, 17], which requires the presence of two or more of the following five criteria: fever, abdominal pain, chest pain, arthritis, and family history of FMF. Patients were followed up at the Pediatric Rheumatology Clinic of the Cairo University Specialized Pediatric Hospital during the period of January 2014 to May 2015.

Inclusion criteria for the study included age of disease onset before 18 years and all patients should have had an attack at least two weeks prior to the time of serum amyloid measurement.

The Cairo University Clinical Research Ethics Committee approved the study protocol, and informed consents were obtained from parents of all patients.

Patients were subjected to detailed history taking, including age at onset of symptoms, age at diagnosis, disease duration, features of attacks (frequency, duration, and clinical manifestations), and dose of colchicine taken by patients. Patients were divided into 2 groups according to the current dose of colchicine: patients on low dose “<1.5 mg/day” and the others on high dose “≥1.5 mg/day”. For all patients duration of colchicine treatment and adherence to colchicine (adherence being defined as no colchicine dose missed in at least the last 6 months before the time of study) were recorded. Colchicine unresponsiveness defined clinically as the occurrence of at least one attack per month despite daily treatment with 2 mg of colchicine or more was also recorded [7, 8].

Patients were subjected to thorough physical examination, and the following laboratory tests were performed: complete blood count (CBC, using blood cell counter, Cell-Dyn 3700; Abbott), CRP (screened by latex agglutination test [18] and confirmed by nephelometry analysis), ESR [19], and urine analysis, besides assessment of serum amyloid A (SAA) level. The results of MEFV gene mutations testing were recorded from the patients' files.

For determination of SAA level, blood samples were withdrawn from patients, left to clot, and then centrifuged at 10000 rpm for 20 minutes. The separated serum was aspirated and stored frozen at −80°C until analysis of SAA, which was done by ELISA kits supplied by Cusabio, USA, according to manufacturer's instruction [20]. Each sample was tested in duplicate and the average was taken. A cutoff level of 30 mg/L SAA level was chosen to reflect a real increase in its level.

Measurement of SAA is usually done in our patients to evaluate disease activity and response to colchicine and to evaluate risk of amyloidosis in patients with frequent attacks despite maximum tolerated dose of colchicine.

### 2.1. Statistical Analysis

Data analysis was performed through Statistical Package of Social Sciences (SPSS) software program for windows version 21. Data were expressed as mean and standard deviation for quantitative variables and number and percentage for qualitative ones. Comparison was performed through Chi-square or Fisher's exact test for qualitative variables and independent sample *t*-test for quantitative ones. Pearson correlation coefficient was calculated to signify the association of quantitative variables. *P* values < 0.05 were considered significant.

## 3. Results

The study included 71 children with FMF, 36 males and 35 females, with a mean age of 9.0 ± 3.7 years. The mean age at disease onset was 5.0 ± 2.9 years, and mean age at disease diagnosis was 7.1 ± 3.4 years. The mean disease duration was 4.0 ± 2.8 years. Six patients (8.5%) had family history of FMF, 6 patients (8.5%) had family history of renal disease, and 25 patients (35.2%) had consanguineous parents. Ten patients (14.1%) had proteinuria.

With regard to the distribution of MEFV gene mutation among the studied patients, 46 patients (64.8%) were heterozygous, 13 patients (18.3%) were compound heterozygous, and 12 patients (16.9%) were homozygous. The allelic frequency of MEFV mutations was V726A (32.4%), M694V (23.9%), M694I (23.9%), E148Q (22.5%), and M680I (15.5%).

All patients were on colchicine therapy, with mean duration of 1.9 ± 1.8 years. Forty-five percent (45%) of patients have low adherence to colchicine therapy, mainly due to diarrhea or social and economic factors, 16% medium adherence and 39% high adherence.

The frequency of attacks in the studied patients was 2.9 ± 2.8/month, with a mean duration of 1.6 ± 1.6 days. The clinical characteristics of FMF patients during attacks are presented in Table 1.

The results of laboratory investigations at the time of study are displayed in Table 2. High SAA levels (≥30 mg/L) were found in 78.9% of patients with a mean ± SD of 81.62 ± 31.6 mg/L. Further, 70.4% of the studied patients had increased ESR, with a mean ± SD of 38.1 ± 16.7 mm/hr, and only 31% had high CRP level, with a mean value ± SD of 15.4 ± 12.8. However, no significant correlation was found between SAA level and either ESR (correlation coefficient −0.105 and *P* value 0.382) or CRP level (correlation coefficient −0.125, with *P* value 0.579).

When stratified according to the level of serum amyloid, whether low (<30 mg/L) or high (≥30 mg/L), no statistically significant correlation could be found between SAA levels and demographic, clinical characteristics and type of gene mutation (Table 3).

It is worth noting that high SAA levels are more frequent in female than in male patients (91.4% and 66.7%, resp.) and are associated with more clinical manifestations during attacks as well as increased ESR and positive CRP. Amongst patients having high SAA levels, 39 patients (39/56, 69.6%) showed increased ESR, and 16 patients out of 22 patients having positive CRP (72.7%) had high SAA.

Further, higher mean SAA levels were more frequent in patients with heterozygous and compound heterozygous patients than the homozygous patients were, though the difference was statistically insignificant.

The relations of SAA level to the dose of colchicine used and duration of treatment as well as adherence to treatment are presented in Table 4.

The mean SAA levels were higher in patients on low colchicine dose compared to patients on high colchicine dose (70.0 ± 37.1 mg/L and 68.4 ± 35.9 mg/L, resp., with *P* value < 0.9), though the differences were statistically insignificant.

## 4. Discussion

The aim of the present study was to measure the SAA levels in Egyptian FMF children two weeks following the last FMF attack, as a marker of subclinical inflammatory process during these periods, correlating the SAA levels with demographic, clinical, and laboratory variables, colchicine dosage, and adherence to treatment.

Seventy-eight percent (78.9%) of the study group had high SAA levels two weeks after the last FMF attack. The results agree with those of Duzova et al. [15] who found SAA levels above the normal range in more than 95% of their studied children with FMF in between the attacks. On the other hand, Lachmann et al. [7] and Berkun et al. [12] reported elevated SAA in only 33.3% and 25% of their FMF patients, respectively, during attack-free periods. The discrepancy between their results and the present findings may be due to the difference in the studied age groups and perhaps to the difference in the compliance of patients to colchicine. Additional factor is the inability to give high therapeutic doses of colchicine in a considerable number of children due to intolerance and occurrence of complications, mainly diarrhea.

Further, CRP was found positive in 31% of patients in the present study which is in concordance with the findings of Duzova et al. (CRP positive in 37.7%) [15] and Korkmaz et al. (CRP positive in 34%) [9]. Also, Lachmann et al. [7] reported that CRP was higher than normal in FMF patients during attack-free periods compared to healthy controls. Yet, no correlation was found between SAA and CRP in the present study, unlike reports of Duzova et al. [15] and Berkun et al. [12] who found high correlation between SAA levels and CRP.

The elevated SAA levels in about 79% of patients and positive CRP in about 1/3 of patients, together with increased ESR in about 2/3 of patients, observed in the present study, point to continuation of the inflammatory process during the attack-free periods. Further, it appears that SAA is a better indicator of the subclinical inflammation.

Moreover, the present findings revealed that high SAA was significantly associated with the female gender. It was more commonly present in children of consanguineous parents and those with family history of FMF and renal disease. Berkun et al. [12] reported that elevated SAA was significantly associated with family history of FMF and high serum CRP but not with age or gender.

In the present work higher SAA values were encountered in patients on low colchicine dose, though the difference was statistically insignificant compared to patients on high colchicine dose. Duzova et al. [15] reported similar findings. These findings indicate that nonresponse to colchicine may be partly due to incompliance due to complications or socioeconomic factors. Effort should be done to improve compliance by minimizing the complications and ensuring regular supply of the medication through health insurance system. The difference may be also attributed to the effect of individual factors on the serum level of the drug compared to the level of colchicine within the granulocytes, determined by p-glycoprotein pump [21]. Moreover, the measurement of SAA can be used to detect patients with poor compliance, which is not always perfect in children with FMF. Different forms of incompliance were reported in our study group; as some patients may stop therapy on their own, others may use less than the recommended colchicine dose once the severity of attacks subsides or the frequency decreases. Moreover, children encounter frequent complications from colchicine mainly diarrhea, so in spite of being adherent to colchicine, full therapeutic dose cannot be reached.

It is, however, worth mentioning that following recapitulation of the present results, the dose of colchicine was increased in some patients with high SAA levels, together with a trial to increase the tolerance by dose splitting and urge patients to adhere to colchicine therapy.

High SAA levels were detected two weeks after last FMF attack in a large percentage of Egyptian FMF children. This indicates that subclinical inflammation continues during attack-free periods, and SAA could be used as a marker of it. Measurement of SAA in a larger number of patients after increasing the colchicine dose and improving the compliance may help in decreasing the risk of amyloidosis.

The high levels of SAA may be also due to incompliance in about 45% of Egyptian FMF children. Effort should be directed towards improving colchicine tolerance and providing regular supply of colchicine for FMF Egyptian children. Further studies to decrease gastrointestinal side effects of colchicine may be beneficial especially in children.

## Figures and Tables

**Table 1:** Clinical characteristics of the studied FMF patients during the FMF attack.

Variables	Frequency
Fever	64 (90.1%)
Abdominal pain	70 (98.6%)
Chest pain	49 (69%)
Myalgia	28 (39.4%)
Arthritis	25 (35.2%)
Arthralgia	53 (74.6%)
Rash	9 (12.7%)
Oral ulcers	17 (23.9%)
Testicular affection	4 (11.1%) (4/36 males)

**Table 2:** Laboratory findings of studied FMF patients at the time of study.

Variable	Range	Mean ± SD
Hemoglobin (gm/dL)	10.1–13.9 (71)	12.3 ± 0.9
Total leucocytic count (×1000/*µ*L)	3.8–23.4 (71)	7.7 ± 3.2
Platelet count (×1000/*µ*L)	134–523 (71)	317 ± 78.2
ESR (mm/hr)		
<15	3–14 (21/71, 29.6%)	9.6 ± 3.4
≥15	15–90 (50/71, 70.4%)	38.1 ± 16.7
CRP Titer		
Positive	2.4–48 (22/71, 31%)	15.4 ± 12.8
Negative	0 (49/71, 69.0%)	
Serum amyloid A (SAA) (mg/L)		
<30 mg/L	13.7–29.5 (15/71, 21.1%)	24.8 ± 4.8
≥30 mg/L	32.6–129.2 (56/71, 78.9%)	81.62 ± 31.6

Number in parentheses is number of patients.

Only 5 patients (7%) had TLC 13.7–23.4 × 10^3^ per microliter of blood.

The median SAA level was 76.1 mg/L (for the range 13.7–129.2 mg/L).

**Table 3:** Demographic and clinical characteristics of studied patients stratified according to the level of SAA.

	Serum amyloid A level (SAA)		*P* value
	Low (<30 mg/L)	High (>30 mg/L)	
Sex			
Male	12/36 (33.3%)	24/36 (66.7%)	0.018
Female	3/35 (8.6%)	32/35 (91.4%)	
Positive consanguinity	5/25 (20%)	20/25 (80%)	1.0
Family history of FMF	2/6 (33.3%)	4/6 (66.7%)	0.6
Family history of renal disease	1/6 (16.7%)	5/6 (83.3%)	1.0
Age at disease onset (year)	4.87 ± 3.04	4.98 ± 2.85	
Age at disease diagnosis (year)	6.98 ± 3.32	7.14 ± 3.43	
Duration between the disease onset and diagnosis (year)	1.90 ± 1.93	2.12 ± 2.17	
Disease duration (year)	3.97 ± 2.54	4.00 ± 2.90	
Age at time of study (year)	8.78 ± 3.60	9.07 ± 3.73	
Frequency of attacks/month	2.27 ± 2.95	3.08 ± 2.82	
Duration of attacks (day)	1.30 ± 0.90	1.65 ± 1.73	
MEFV mutation (HH)			
Heterozygous	10/46 (21.7%)	36/46 (78.3%)	1.0
Compound heterozygous	2/13 (15.4%)	11/13 (84.6%)	0.72
Homozygous	3/12 (25%)	9/12 (75%)	0.71
Fever	13/64 (20.3%)	51/64 (79.7%)	0.63
Abdominal pain	15/70 (21.4%)	55/70 (78.6%)	1.0
Chest pain	10/49 (20.4%)	39/49 (79.6%)	1.0
Myalgia	4/28 (14.3%)	24/28 (85.7%)	0.37
Arthritis	8/25 (32%)	17/25 (68%)	0.13
Arthralgia	9/53 (17%)	44/53 (83.01%)	0.18
Rash	2/9 (22.2%)	7/9 (77.8%)	1.0
Testicular affection	2/4 (50%)	2/4 (50%)	0.59
Proteinuria	3/10 (30%)	7/10 (70%)	0.43
ESR (≥15 mm/Hg)	11/50 (22%)	39/50 (78%)	
+ve CRP	6/22 (27.3%)	16/22 (72.7%)	0.53

**Table 4:** Correlation between colchicine treatment and SAA level.

Colchicine treatment	SAA level	
	Low level (<30 mg/L) (*n* = 15)	High level (≥30 mg/L) (*n* = 56)
Duration of colchicine treatment	2.0 ± 1.56 years	1.86 ± 1.83 years
Colchicine dosage		
Low dose (<1.5 mg/day)	10 patients (18.9%)	43 patients (81.1%)
High dose (≥1.5 mg/day)	5 patients (27.8%)	13 patients (72.2%)
Adherence to colchicine	7 patients (18%)	32 patients (82%)

Note: (*n*) = number of patients.
